# Effects of light acclimation on shoot morphology, structure, and biomass allocation of two *Taxus* species in southwestern China

**DOI:** 10.1038/srep35384

**Published:** 2016-10-13

**Authors:** Wande Liu, Jianrong Su

**Affiliations:** 1 Research Institute of Resources Insects, Chinese Academy of Forestry, Kunming 650224, China; 2 Pu’er Forest Eco-system Research Station, China’s State Forestry Administration, Kunming 650224, China

## Abstract

Acclimation to changing light conditions plays a crucial role in determining the competitive capability of tree species. There is currently limited information about acclimation to natural light gradient and its effect on shoot structure and biomass in *Taxus* species. We examined the acclimation of the leaf and shoot axis morphology, structure and biomass allocation of *Taxus yunnanensis* and *T. chinensis* var. *mairei* under three different natural light environments, full daylight, 40–60% full daylight and <10% full daylight. The leaf biomass, nitrogen content per unit area, leaf carbon content per dry mass and leaf dry mass to fresh mass ratio increased with light in both species, demonstrating an enhanced investment of photosynthetic biomass and structural investment under high light. The number of leaves per unit shoot axis length and the leaf dry mass per unit shoot axis length increased with light in both species. However, the light increase did not result in the increase of the total shoot mass. *T. yunnanensis* produced larger leaves under low light and a higher shoot axis length per unit dry mass under high light, whereas the leaf size and biomass yield of *T. chinensis* var. *mairei* were not sensitive to light.

Acclimation to changing light conditions typically plays a crucial role in determining the competitive capacity of forest tree species^1^,^2^. In most forests, light is one of the most limiting resources for plant growth and survival^3^. Light conditions change over time. Changes in sun flecks occur within minutes, whereas changes in canopy gap closure occur on a time scale of years^4^. Plant performance is enhanced through morphological, physiological and structural acclimation to the light environment^3^,^5^,^6^.

Acclimation is achieved through adjustments at both the leaf and shoot axis levels^7^. Leaf adaptations to light include changes in leaf morphology, physiology and structure such as sun leaves. Sun leaves, which grow in exposed conditions of the canopy, are thicker, smaller or more slender and have a greater mass per area^8^, a higher volume of photosynthetic machinery per unit leaf area^9^ and higher growth rates^10^ than shade leaves. In addition, sun leaves have higher nitrogen content per unit area^8^. In contrast, shade leaves, growing under the canopy, have a lower nitrogen content, which reduces respiration costs, a higher specified leaf area (SLA), which increases the efficiency of light capture and a higher chlorophyll content^11^ compared with sun leaves. Shade leaves are thin and less hardy, because wind and desiccation stresses are lower in the understory. Sun leaves tend to be arranged approximately cylindrically around shoots, whereas shade leaves tend to be flat and horizontal^12^. The main consequence of these changes in leaf morphology seems to be able to harvest light more efficiently^13^.

The adaptation of the shoot axis to light conditions primarily affects the size and patterns of the shoot axis. The shoot axis provides mechanical support and a hydraulic pathway for leaves^14^. A field survey has shown that the shoot axis developed under high-light conditions tend to be shorter than shade-developed shoot axis; whereas the shoot axis length might influence the numbers of leaves on the shoot^13^. Shoot patterns typically affect tree structure^12^. Tree structure characteristics are closely associated with light interception and photosynthetic production^15^. Shoot patterns of trees, therefore, have frequently been associated with light demand^12^,^16^.

Acclimation to changing light conditions can be achieved through adjustments in biomass allocation to various organs, such as leaves and the shoot axis^14^,^17^. The relative amount of biomass present in the various organs is not fixed but can vary over time across environments. For example, in low irradiance conditions, shade-tolerant plants enhance the interception of light with a large leaf area per unit leaf biomass^3^ and shade leaves enhance the potential relative growth rates through little physiological activity (slow respiration and light-saturated photosynthetic rates). Moreover, shoots under high irradiance conditions have a circular cross section with tightly packed leaves^18^,^19^, thus maximizing foliar biomass investments per unit shoot length and taking advantage of the increased irradiance for photosynthesis^13^. A quantitative understanding of biomass allocation patterns is of fundamental importance to plant ecology and evolution and is useful for agricultural and forest practices and implementation^20^.

*Taxus* species are well-known for their high content of taxol, which exhibits bioactivities against certain tumors^21^,^22^. Although most *Taxus* species are protected plants in many areas and many studies have been conducted on their pollination biology^23^, genetics^24^ and taxol production^25^, few studies have characterized the changes exhibited by *Taxus* species under different light conditions^26^,^27^, and little research has been conducted on their shoot biomass allocation along a natural light gradient and the effects of light on the shoot structure of *Taxus* species are not well addressed^22^. In the present study, the acclimation of shoot structure and biomass allocation in response to a natural light gradient for two *Taxus* species, *Taxus yunnanensis* and *T. chinensis* var. *mairei*, was investigated to determine the contributions of leaf- and shoot-level adjustments in structure and biomass allocation, and to characterize the potential role of shoot structure on species competitiveness. Specifically, this study addressed (1) how shoot morphology and structure are acclimated to light environments and (2) how shoot biomass is allocated under different light environments.

## Results

### Leaf size and morphology along a natural light gradient

**Figure 1:**
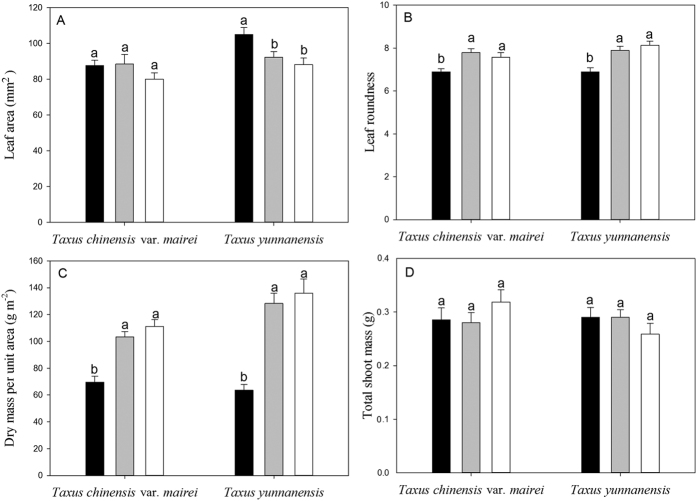
Bar charts (mean ± SE) of leaf area (**A**) leaf roundness (leaf length/leaf width, **B**) leaf dry mass per unit area (**C**) and total shoot mass (leaf + shoot axis dry mass, **D**) for *Taxus chinensis* var. *mairei* and *Taxus yunnanensis* along a natural light gradient. Solid black bars represent low light, gray bars represent middle light, and white bars represent high light. Different letters (a,b) indicate significant differences.

Individual leaf area (*A*_L_) decreased with increasing light levels for *T. yunnanensis* but not for *T. chinensis* var. *mairei* (Fig. 1A). Leaf roundness (Fig. 1B) and leaf biomass per unit area (LMA, Fig. 1C) increased with increasing light levels in both *T. yunnanensis* and *T. chinensis* var. *mairei*.

*T. yunnanensis* had a higher *A*_L_ in low light and LMA in middle and high light compared with *T. chinensis* var. *mairei* (*P* < 0.05). Leaf roundness did not differ between *T. yunnanensis* and *T. chinensis* var. *mairei* under all light conditions (*P* > 0.05).

### Changes in foliar nitrogen and structural investments along a natural light gradient

**Figure 2:**
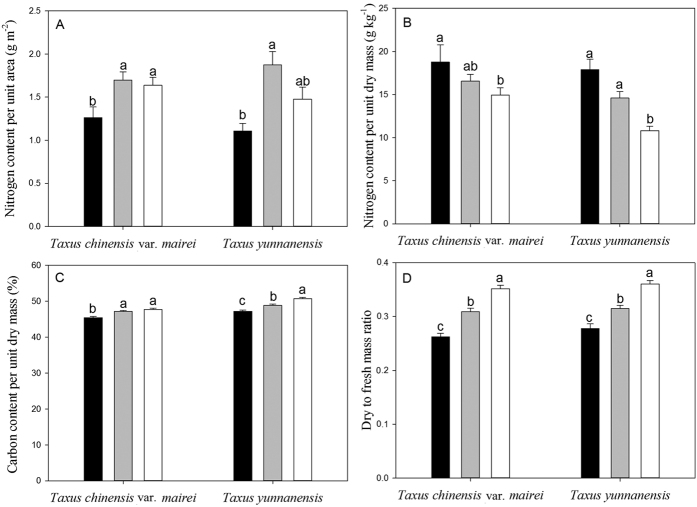
Bar charts (mean ± SE) of nitrogen content per area (**A**) nitrogen content per dry mass (**B**) carbon content per dry mass (**C**) and leaf dry to fresh mass ratio (**D**) for *Taxus chinensis* var. *mairei* and *Taxus yunnanensis* along a natural light gradient. Solid black bars represent low light, gray bars represent middle light, and white bars represent high light. Different letters (a,b) indicate significant differences.

The leaf nitrogen content per unit area (*N*_area_, Fig. 2A), leaf carbon content per unit dry mass (Fig. 2C) and leaf dry mass to fresh mass ratio (Fig. 2D) increased with light in both species. Overall, the total leaf carbon content per unit dry mass and the dry to fresh mass ratio were strongly correlated with the light level, thus suggesting that both variables reflect changes in leaf structural investments. In contrast with *N*_area_, the leaf nitrogen content per unit dry mass (*N*_mass_) decreased with increasing light in *T. yunnanensis* and *T. chinensis* var. *mairei* (Fig. 2B).

*T. yunnanensis* had lower *N*_mass_ under high light and higher leaf carbon content per unit dry mass under all light conditions compared with *T. chinensis* var. *mairei* (*P* < 0.05). However, there were no significant differences in *N*_area_ and the dry to fresh mass ratio between *T. yunnanensis* and *T. chinensis* var. *mairei* under all light conditions (*P* > 0.05).

### Changes in shoot axis morphology and biomass along a natural light gradient

**Figure 3:**
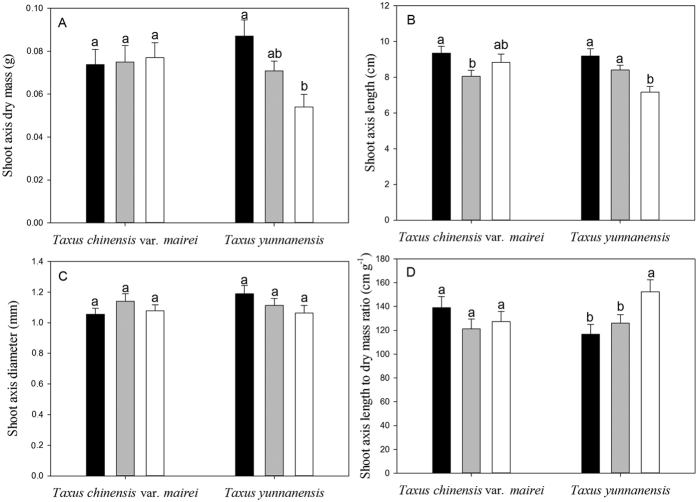
Bar charts (mean ± SE) of shoot axis dry mass (**A**) shoot axis length (**B**) shoot axis diameter (**C**) and shoot axis length to dry mass ratio (**D**) for *Taxus chinensis* var. *mairei* and *Taxus yunnanensis* along a natural light gradient. Solid black bars represent low light, gray bars represent middle light, and white bars represent high light. Different letters (a,b) indicate significant differences.

Shoot axis dry mass decreased with light in *T. yunnanensis* (Fig. 3A), whereas shoot axis length per unit dry mass increased with light (Fig. 3D). Shoot axis dry mass and shoot axis length per unit dry mass did not differ among different natural light conditions in *T. chinensis* var. *mairei* (*P* > 0.05) (Fig. 3A,D). Shoot axis length decreased with light in both *T. yunnanensis* and *T. chinensis* var. *mairei* (Fig. 3B). Shoot axis diameter was not sensitive to light in either *T. yunnanensis* or *T. chinensis* var. *mairei* (Fig. 3C).

Only under high light did a significant difference exist between *T. yunnanensis* and *T. chinensis* var. *mairei* in shoot axis dry mass and shoot axis length (*P* < 0.05). The shoot axis diameter and shoot axis length per unit dry mass did not differ between *T. yunnanensis* and *T. chinensis* var. *mairei* under any of the three light conditions (*P* > 0.05).

### Biomass allocation between leaf and shoot axis along a natural light gradient

The total shoot mass was not associated with light in either species (Fig. 1D). However, the number of leaves per unit shoot axis length and leaf dry mass per unit shoot axis length increased with increasing light in both *T. yunnanensis* and *T. chinensis* var. *mairei* (Table 1). The leaf dry mass per unit shoot axis dry mass, the number of leaves per unit shoot axis dry mass and leaf dry mass per unit total shoot mass also increased with light in *T. yunnanensis*, but not in *T. chinensis* var. *mairei* (Table 1). The leaf area per unit total shoot mass did not differ among the three light conditions in *T. chinensis* var. *mairei* (*P* > 0.05). *T. yunnanensis* had the highest leaf area per unit total shoot mass under high light (*P* < 0.05).

The total shoot mass and leaf dry mass per unit shoot axis length did not differ between *T. yunnanensis* and *T. chinensis* var. *mairei* under any of the three light conditions (*P* > 0.05), and there were no significant differences between *T. yunnanensis* and *T. chinensis* var. *mairei* in the number of leaves per unit shoot axis length, leaf dry mass per unit shoot axis dry mass, number of leaves per unit shoot axis dry mass, leaf dry mass per unit total shoot mass or leaf area per unit total shoot mass, except for under high light. *T. yunnanensis* had a higher number of leaves per unit shoot axis length, leaf dry mass per unit shoot axis dry mass, number of leaves per unit shoot axis dry mass, leaf dry mass per unit total shoot mass and leaf area per unit total shoot mass compared with *T. chinensis* var. *mairei* under high light (*P* < 0.05).

## Discussion

### Adjustment of leaf structure and chemistry to a natural light gradient

In plants, phenotypic plasticity may be expressed at various levels, such as the shoot or leaf level; as a result, in trees, the morphological and physiological properties of leaves dramatically change in response to light gradients^18^,^28^,^29^. In the present study, smaller leaves were observed under *T. yunnanensis* in high light and longer leaves were observed for both *Taxus* species under high light. Decreases in the mean area of individual leaves have been frequently observed as light increases^13^,^30^,^31^,^32^ but not consistently^33^. Smaller leaves improve the coupling between the leaves and the atmosphere, thereby reducing leaf temperature and transpiration^13^. Leaf coupling with the atmosphere is further enhanced through increased leaf elongation, thus decreasing the effective leaf size for a common leaf area^13^. In addition, smaller leaves increase the homogeneity of the radiation field and enhance the penetration of the canopy through penumbral radiation^34^. Although the leaves became smaller under high light, the number of leaves per unit shoot axis length was positively correlated with light for the two species (Table 1).

LMA is an important index of leaf structure that is highly correlated with the light environment and is closely associated with photosynthesis^35^. The results of the present study showed a significant increase in LMA with increasing light in the two species, similarly to findings in other studies^1^,^13^,^27^,^35^,^36^. A greater variation in LMA suggests that LMA is an important factor in the acclimation of these two species to various light conditions. Higher LMA suggests that there is more tissue in which photosynthetic reactions can occur when the light strikes the leaves. Lower and higher LMA leaves were able to use light efficiently in high light^37^. Smaller leaves have fewer cells with thicker cell walls and show lower quantum efficiency^38^; therefore, smaller leaves may reduce heat loading. Leaves become smaller as light levels increase, thus leading to increased LMA^37^. A lower LMA in lower-light situations has been proposed to be an important adaptive characteristic allowing species to construct a larger foliar area with a given fraction of plant mass in the leaves^39^. In a simulation study, Sims *et al*.^40^ have shown that growth is highly dependent on adjustments in LMA to low and high light.

The needle *N*_area_ increased with increased light for both *Taxus* species in the present study, reflecting the much higher variability in LMA than *N*_mass_. The close relationship between leaf *N*_area_ and light level has previously been reported^13^,^41^,^42^,^43^ and reflects the increase in the carboxylating capacity of sun-adapted leaves mediated through an increase in the Rubisco-associated nitrogen pool^41^. In contrast, the needle *N*_mass_ decreased with increasing light for both *Taxus* species. Several studies have shown that the *N*_mass_ either increases or decreases with light within tree or forest canopies. For example, the leaf or needle nitrogen concentration on a dry weight basis has been positively correlated with relative irradiance in several species, such as *Eucalyptus grandis*^44^ and *Betula pendula*^43^. In contrast, the *N*_mass_ decreases with increasing leaf or needle irradiance for the shrubs *Corylus avellana* and *Lonicera xylosteum*^43^, *Fagus sylvatica*^45^, *Acer platanoides* and *Padus avium*^46^, and *Picea abies*^1^,^47^, and this pattern reflects the different light demands of these species^42^,^43^. Thus, the results of the present study are consistent with the conclusion that *N*_mass_ decreases with increasing leaf or needle irradiance in shade-intolerant species, such *Picea abies*. Moreover, many physiological processes, such as photosynthesis and growth, are closely correlated with *N*_mass_, because photosynthate production and translocation exhibit different time lags.

Leaf carbon content per dry mass and leaf dry mass to fresh mass ratio were also positively correlated with light, suggesting greater structural investments under high light. Greater structural investments in leaves acclimated to higher light enhance leaf rigidity and may improve leaf resistance to low leaf water potentials^48^, but these investments might also reduce leaf photosynthetic capacity per unit dry mass^49^. Under low light, the moisture conditions and lower levels of evaporation facilitate high leaf water content, thus leading to a lower leaf dry mass to fresh mass ratio, and maintenance of turgor, and providing a low-resource method to enhance leaf display for light capture. Leaf carbon content per dry mass and leaf dry mass to fresh mass ratio increased as the light intensity increased, leading to increased leaf mass. The variation in leaf carbon content per dry mass and leaf dry mass to fresh mass ratio under different light conditions may contribute to functional and ecological segregation^38^.

### Acclimation of shoot axis morphology to natural light gradient

In the present study, the shoot axis length decreased with increasing light. Küppers, using hedgerow species, has elegantly demonstrated differences between tree species in shoot growth in response to a heterogeneous light environment^50^. The study has also shown that sun-shoots have shorter shoot axis lengths and are less efficient in expansion into potentially unoccupied space and prevention of self-shading compared with shade-shoots. These findings are consistent with a typical foraging mechanism. According to the foraging theory, the shoot axis should be shorter, and branching frequency should be higher in branches exposed to higher resource availability. On the one hand, species reduce the shoot length under high light. In this way, smaller masses of woody support tissue are invested in the production of unit leaf tissue. The advantage of a smaller support mass is that more biomass is available for new leaf growth. This shift in biomass allocation from stem to leaf mass improves competitive capability. At the same time, relatively long shoots develop under low light, thus enabling expansion into potentially unoccupied spaces and preventing self-shading. On the other hand, species also occupy more space and efficiently intercept more light through increased branching frequency. This phenomenon reflects a capability to sense neighbors through phytochrome-based mechanisms^12^.

In contrast to other plant resources, light availability depends not only on leaf area but also on the shoot axis length. Longer shoots occupy more space and intercept more light under low light. Some studies have shown that maximum shoot growth occurs in shady conditions^51^. The opposite growth pattern has also been observed in some studies, showing that shoots are often shorter under lower light^52^,^53^. The adjustments of shoot axis length provide an explanation for the greater efficiency of light interception in plants grown in low light relative to open fields^54^.

Unlike the shoot axis length, the shoot axis diameter was not sensitive to light in the present study. There were no significant differences among three light environments for either species. The responses of shoot axis diameter to a heterogeneous light environment indicated that diameter growth is not a function of the acclimation of shoot axis morphology. Increased diameter growth under high light reflected only an enhanced investment of shoot axis biomass, which in turn reduced the investment of leaf biomass under steady total shoot mass and weakened photosynthesis and competitive capacity.

### Acclimation of biomass allocation to a natural light gradient

Decreased light availability changed the allocation to shoot-level biomass of *Taxus* species. Although total shoot mass was not associated with light in *T. yunnanensis* and *T. chinensis* var. *mairei*, the number of leaves per unit shoot axis length and leaf dry mass per unit shoot axis length increased with increasing light in both species, suggesting that more biomass was allocated to the leaves. In high-light environments, light dispersed over more leaves is used more efficiently in photosynthesis and less is lost to saturation^55^, whereas in low-light environments, light concentrated on a few leaves is efficiently intercepted at a minimal cost in leaf construction and maintenance^56^.

In contrast, the changes in leaf area per unit total shoot mass did not show a clear trend for either species, although *T. yunnanensis* had the highest leaf area per unit total shoot mass under high light. However, the leaf dry mass per unit shoot axis dry mass, number of leaves per unit shoot axis dry mass and leaf dry mass per unit total shoot mass were positively correlated with light in *T. yunnanensis* but not in *T. chinensis* var. *mairei*. These results showed that less biomass was invested in woody support tissue in *T. yunnanensis*. In this way, more new production of a unit leaf tissue can be produced at the risk of an increased self-shading of the crown. This shift in biomass allocation from shoot axis to leaf mass is also reflected in the high photosynthesizing tissue of understory saplings^3^.

### Species differences in plastic adjustment to a natural light gradient

Although the species examined in the present study are within the same genera, significant differences existed in shoot structure and biomass allocation adjustments to natural light gradients. *T. yunnanensis* had larger leaves under low light, greater LMA under moderate and high levels of light, higher leaf carbon content per unit dry mass under all light conditions, and lower leaf *N*_mass_ under high light compared with *T. chinensis* var. *mairei*. All the above-mentioned leaf characteristics reflect changes in light interception efficiency, investment photosynthetic biomass, and leaf structural investments. Variations in leaf ecophysiology among species in different taxa have been studied to understand acclimation to light^13^,^32^,^57^, but few studies have addressed the leaf changes within the same genera^28^. The results of the present study demonstrated that even within the same genera, species exhibit distinct ecophysiological characteristics to acclimate to light. Larger leaves under low light suggest that more diffuse or direct light can be intercepted. Greater LMA under high light suggests that when this light strikes sun leaves, there will be more tissue in which photosynthetic reactions can occur. Accordingly, a higher leaf carbon content per unit dry mass shows that *T. yunnanensis* had greater structural investments under high light.

Except for leaf morphological and physiological adjustments, *T. yunnanensis* exhibited a lower shoot axis dry mass and shoot axis length, with a higher number of leaves per unit shoot axis length, leaf dry mass per unit shoot axis dry mass, number of leaves per unit shoot axis dry mass, leaf dry mass per unit total shoot mass and leaf area per unit total shoot mass compared with *T. chinensis* var. *mairei* under high light. These characteristics suggest that more biomass is allocated to the leaves in *T. yunnanensis*. Higher leaf biomass increased carbon fixation and biomass growth and potential relative growth rates. Differences in leaf and shoot axis morphology and biomass allocation may substantially modify the competitive potential of a species. The results of the present study indicate that *T. yunnanensis* is more competitive than *T. chinensis* var. *mairei* in the same light environment.

## Conclusions

Positive correlations among leaf biomass, nitrogen content per unit area and light indicated an enhanced investment of photosynthetic biomass under high light and a greater leaf-level light-harvesting efficiency. The leaf carbon content per dry mass and leaf dry mass to fresh mass ratio of all species were also correlated positively with light, thus suggesting a greater structural investment under high light. Although increased light did not result in an increase in the total shoot mass, the shoot axis dry mass decreased with increasing light levels and enhanced the biomass yield in leaves and the investment of photosynthetic biomass. These structural acclimations showed that increased light resulted in the increase of the investment in photosynthetic biomass.

Species differences in structural acclimation demonstrated different foliar investment of photosynthetic biomass. Larger leaves resulted in more efficient harvesting of diffuse irradiance under low light in *T.yunnanensis*. In addition, the shoot axis length per unit dry mass, leaf dry mass per unit shoot axis dry mass, leaf number per unit shoot axis dry mass and leaf dry mass per unit total shoot mass increased with increasing light in *T. yunnanensis*, suggesting an enhanced investment of photosynthetic biomass under high light. These structural acclimations indicated that interspecific variations in shoot architectural adjustments may play an important role in differentiating species-specific photosynthetic potential.

## Materials and Methods

### Study species and sites

The field experiments were conducted at Jingdong Station (23°56′–24°50′N, 100°21′–101°15′E) of the Research Institute of Resources Insect, Chinese Academy of Forestry (CAF) in Yunnan Province, southeastern China. The elevation of this field station is 1200 m a.s.l. The mean annual temperature is 18.3 °C, with a range from −2 °C in January to 37 °C in July^22^. The mean annual precipitation is 1100 mm with a seasonal distribution, primarily occurring in summer (June-September)^22^. Two *Taxus* species (*Taxus yunnanensis* and *T. chinensis* var. *mairei*) were planted by hand in 2008 under three light environments. We evaluated the shoot biomass allocation under three different natural light conditions: full daylight (high light), 40–60% full daylight (middle light) and <10% full daylight (low light), estimated at mid-day using an LI-190SA quantum sensor (Li-Cor, Inc., Lincoln, NE, USA)^22^. The study locations were in proximity to the mean distance between sites 27 m (24–33 m). The soil in the sampled sites was latosol. Soil samples were collected under all three light conditions and subsequently tested, and there were no significant differences among the sites in terms of soil organic matter, N, P and K content, or available nutrients. This work was conducted in accordance with the forestry standards of the “Observation Methodology for Long-term Forest Ecosystem Research” of the People’s Republic of China.

### Plant materials and data collection

Terminal shoots (including leaves and twigs) were all sampled under each of the three different natural light conditions. Under each natural light condition, we also randomly selected 20 individuals of similar height (188.6 ± 11.7 cm), and collected three terminal shoots from the top of each individual for analysis. Sixty individuals were sampled from each species, with 360 terminal shoots collected for analysis.

Sampled shoots were removed from trees, stored in plastic bags and transported to the laboratory in a cooling box. The number of leaves per a shoot were counted in the laboratory; the leaf fresh weight was measured with an electronic balance (±0.0001 g); and leaf length, leaf width and leaf area were measured with a portable area meter (AM-300). The leaves were dried at 85 °C to a constant weight and then weighed to determine the leaf dry mass. All leaves from the same individual were pooled for chemical analysis. Leaf nitrogen (mass basis, *N*_mass_) was measured using an elemental analyzer (NCS2500, Carlo Erba Instruments, Milan, Italy). Leaf carbon content was determined using a vario Macro Elemental Analyzer (Elementar Analysensysteme GmbH, Germany)^58^.

The basal and apical diameters of the shoot axis were measured (using a digital caliper with precision of 0.01 mm) in two perpendicular directions, and a mean was calculated. The shoot axis length (digital caliper with precision of 0.01 mm) and shoot axis dry mass (after drying to a constant mass at 85 °C) were also measured and recorded.

From the collected data, the following parameters were derived: individual leaf area, leaf roundness, leaf dry mass per unit area (LMA), total shoot mass, area-based leaf nitrogen content, shoot axis diameter, shoot axis length per unit shoot axis dry mass, number of leaves per unit shoot axis length, number of leaves per unit shoot axis shoot axis dry mass, leaf mass per unit shoot axis length, leaf dry mass per unit shoot dry mass, and leaf area. The individual leaf area of the shoot was calculated by dividing the total leaf area by the total number of shoot leaves; the leaf roundness was calculated by dividing the leaf length by the leaf width; the LMA was quantified as the ratio of dry leaf weight to the fresh leaf area; the total shoot mass was the sum of shoot axis mass and leaf mass; the area-based leaf nitrogen content was inter-converted via LMA (*N*_area_ = *N*_mass_LMA)^13^,^59^; the shoot axis diameter was considered to be the mean of the basal and apical diameters of the shoot axis; the shoot axis length per unit shoot axis dry mass was quantified as the ratio of shoot axis length to shoot axis dry mass; the number of leaves per unit shoot axis length and the number of leaves per unit shoot axis dry mass were calculated by dividing the number of leaves by shoot axis length and shoot axis dry mass, respectively; the leaf dry mass per unit shoot axis length and leaf dry mass per unit shoot axis dry mass were determined as the ratio of leaf dry mass to shoot axis length and shoot axis dry mass, respectively; the leaf dry mass per unit total shoot mass was quantified as the ratio of leaf dry mass to the total shoot mass; and the leaf area per unit total shoot mass was quantified as the ratio of leaf area to total shoot mass.

### Statistical analysis

A non-parametric analysis was conducted on leaf dry mass per unit area, leaf nitrogen content per unit area, leaf nitrogen content per unit mass, leaf number per unit shoot axis dry mass, leaf dry mass per unit total shoot mass and leaf area per unit total shoot mass for *T. yunnanensis* and leaf nitrogen content per unit mass for *T. chinensis* var. *mairei*. The Kruskal-Wallis analysis of variance with multiple comparisons was used, because the variance was not homogenous among different natural light conditions. For the other above-mentioned parameters, one-way ANOVA with a post hoc Tukey HSD test was used to compare parameter means among different natural light conditions. The independent-samples *t*-test was applied to examine species differences in terms of the parameter means among natural light conditions. All statistical analyses were performed using SPSS17.0 (SPSS Inc.). All tests were performed at a significance level of α = 0.05.

## Additional Information

**How to cite this article**: Liu, W. and Su, J. Effects of light acclimation on shoot morphology, structure, and biomass allocation of two *Taxus* species in southwestern China. *Sci. Rep.* **6**, 35384; doi: 10.1038/srep35384 (2016).

## Figures and Tables

**Table 1:** Mean (±SE) of the number of leaves per unit shoot axis length, leaf dry mass per unit shoot axis length, leaf to shoot axis dry mass ratio, number of leaves per unit shoot axis dry mass, leaf dry mass per unit total shoot mass and leaf area per unit total shoot mass for *Taxus chinensis* var*. mairei* and *Taxus yunnanensis*.

Indexes	Species	Low light	Middle light	High light
Leaf number per unit shoot axis length	*Taxus chinensis* var. *mairei*	3.54 ± 0.13a	3.21 ± 0.12a	3.95 ± 0.17b
	*Taxus yunnanensis*	3.29 ± 0.16a	3.40 ± 0.11a	5.03 ± 0.18b
Leaf dry mass per unit shoot axis length	*Taxus chinensis* var. *mairei*	0.023 ± 0.002a	0.026 ± 0.001ab	0.028 ± 0.002b
	*Taxus yunnanensis*	0.022 ± 0.001a	0.026 ± 0.002b	0.028 ± 0.001b
Leaf dry mass per unit shoot axis dry mass	*Taxus chinensis* var. *mairei*	3.08 ± 0.27a	2.98 ± 0.20a	3.44 ± 0.27a
	*Taxus yunnanensis*	2.58 ± 0.25a	3.32 ± 0.27b	4.17 ± 023c
Leaf number per unit shoot axis dry mass	*Taxus chinensis* var. *mairei*	500.24 ± 41.12a	391.70 ± 31.28b	512.04 ± 43.99a
	*Taxus yunnanensis*	386.60 ± 31.76a	436.41 ± 34.31a	795.14 ± 78.62b
Leaf dry mass per unit total shoot mass	*Taxus chinensis* var. *mairei*	0.74 ± 0.02a	0.74 ± 0.01a	0.76 ± 0.01a
	*Taxus yunnanensis*	0.70 ± 0.02a	0.75 ± 0.01a	0.80 ± 0.01b
Leaf area per unit total shoot mass	*Taxus chinensis* var. *mairei*	1.08 ± 0.07a	0.89 ± 0.09a	0.94 ± 0.08a
	*Taxus yunnanensis*	1.14 ± 0.08ab	0.93 ± 0.05a	1.30 ± 0.10b

Different letters in the same row indicate significant differences.

## References

[b1] Grassi G. & Bagnaresi U. Foliar morphological and physiological plasticity in *Picea abies* and *Abies alba* saplings along a natural light gradient. Tree Physiology 21, 959–967 (2001). 11498343 10.1093/treephys/21.12-13.959

[b2] Cao Y., Zhou B., Chen S., Xiao J. & Wang X. The photosynthetic physiological properties of *Illcium lanceolatum* plants growing under different light intersity conditions. African Journal of Agricultural Research 6, 5736–5741 (2011).

[b3] Poorter L. Light-dependent changes in biomass allocation and their importance for growth of rain forest tree species. Functional Ecology 15, 113–123 (2001).

[b4] Gundersen P., Callesen I. & de Vries W. Nitrate leaching in forest ecosystems is controlled by forest floor C/N ratio. Environmental Pollution 102, 403–407 (1998).

[b5] Zhu S. D., Li R. H., Song J., He P. C., Liu H., Berninger F. & Ye Q. Different leaf cost–benefit strategies of ferns distributed in contrasting light habitats of sub-tropical forests. Annals of Botany 87, 3046–3054 (2015). 10.1093/aob/mcv179 PMC4765538 26684751

[b6] Pegueropina J. J., Sisó S., Sanchoknapik D., Díazespejo A., Flexas J., Galmés J. & Gilpelegrín E. Leaf morphological and physiological adaptations of a deciduous oak (*Quercus faginea* Lam.) to the Mediterranean climate: a comparison with a closely related temperate species (*Quercus robur* L.). Tree Physiology 36, 287–299 (2016). 26496958 10.1093/treephys/tpv107 PMC4885939

[b7] Pegueropina J. J., Sanchoknapik D., Flexas J., Galmés J., Niinemets Ü. & Gilpelegrín E. Light acclimation of photosynthesis in two closely related firs (*Abies pinsapo* Boiss. and *Abies alba* Mill.): the role of leaf anatomy and mesophyll conductance to CO_2_. Tree Physiology 36, 300–310 (2016). 26543153 10.1093/treephys/tpv114 PMC4885940

[b8] Rozendaal D. M. A., Hurtado V. H. & Poorter L. Plasticity in leaf traits of 38 tropical tree species in response to light; relationships with light demand and adult stature. Functional Ecology 20, 207–216 (2006).

[b9] Elser J. J., Dobberfuhl D. R., MacKay N. A. & Schampel J. H. Organism size, life history, and N:P stoichiometry: towards a unified view of cellular and ecosystem processes. BioScience 46, 674–684 (1996).

[b10] Freschet G. T. . Global to community scale differences in the prevalence of convergent over divergent leaf trait distributions in plant assemblages. Global Ecology and Biogeography 20, 755–765 (2011).

[b11] Poorter H. & Nagel O. The role of biomass allocation in the growth response of plants to different levels of light, CO_2_, nutrients and water: a quantitative review. Australian journal of plant physiology 27, 595–607 (2000).

[b12] Kull O. & Tulva I. Shoot structure and growth along a vertical profile within a *Populus - Tilia* canopy. Tree Physiology 22, 1167–1175 (2002). 12414376 10.1093/treephys/22.15-16.1167

[b13] Niinemets Ü., Cescatti A. & Christian R. Constraints on light interception efficiency due to shoot architecture in broad-leaved Nothofagus species. Tree Physiology 24, 617–630 (2004). 15059762 10.1093/treephys/24.6.617

[b14] Poorter H. . Biomass allocation to leaves, stems and roots: meta-analyses of interspecific variation and environmental control. New Phytologist 193, 30–50 (2012). 22085245 10.1111/j.1469-8137.2011.03952.x

[b15] Valladares F. & Pearcy R. W. The functional ecology of shoot architecture in sun and shade plants of *Heteromeles arbutifolia* M. Roem., a Californian chaparral shrub. Oecologia 114, 1–10 (1998). 10.1007/s004420050413 28307546

[b16] Canham C. D., Finzi A. C., Pacala S. W. & Burbank D. H. Causes and consequences of resource heterogeneity in forests: interspecific variation in light transmission by canopy trees. Canadian Journal of Forest Research 24, 337–349 (1994).

[b17] Jarčuška B. & Milla R. Shoot-level biomass allocation is affected by shoot type in *Fagus sylvatica*. Journal of Plant Ecology 16, 1–7 (2012).

[b18] Cescatti A. & Zorer R. Structural acclimation and radiation regime of silver fir (*Abies alba* Mill.) shoots along a light gradient. Plant, Cell & Environment 26, 429–442 (2003).

[b19] Niinemets Ü., Cescatti A., Lukjanova A., Tobias M. & Truus L. Modification of light-acclimation of *Pinus sylvestris* shoot architecture by site fertility. Agricultural and Forest Meteorology 111, 121–140 (2002).

[b20] Reich P. B., Weisel Y., Eshel A. & Kafkafi U. In Plant roots, the hidden half (eds Waisel Y., Eshel A. & Kafkafi U.) 205–220 (Maicel Dekker, 2002).

[b21] Kingston D. & Newman D. Taxoids: cancer-fighting compounds from nature. Current opinion in drug discovery & development 10, 130 (2007). 17436548

[b22] Liu W., Li S., Su L. & Su J. Variation and correlations of leaf traits of two *Taxus* species with different shade tolerance along the light gradient. Polish Journal of Ecology 61, 329–339 (2013).

[b23] Mercuri A. M., Torri P., Casini E. & Olmi L. Climate warming and the decline of *Taxus* airborne pollen in urban pollen rain (Emilia Romagna, northern Italy). Plant Biology 15, 70–82 (2013). 22776105 10.1111/j.1438-8677.2012.00624.x

[b24] Miao Y. C., Lang X. D., Zhang Z. Z. & Su J. R. Phylogeography and genetic effects of habitat fragmentation on endangered *Taxus yunnanensis* in southwest China as revealed by microsatellite data. Plant Biology 16, 365–374 (2014). 23890056 10.1111/plb.12059

[b25] Wang J. W., Zheng L. P. & Tan R. X. Involvement of nitric oxide in cerebroside-induced defense responses and taxol production in *Taxus yunnanensis* suspension cells. Applied Microbiology and Biotechnology 75, 1183–1190 (2007). 17375294 10.1007/s00253-007-0927-7

[b26] Li W., Zhao Y. S., Zhou Z. Q. & Liu T. Comparison of photosynthetic and fluorscence characteristics of leaves in *Taxus cuspidata* under different light intensities. Nonwood Forest Research 30, 51–56 (2012).

[b27] Iszkuło G. Success and failure of endangered tree species: low temperatures and low light availability affect survival and growth of european yew (*Taxus baccata* L.) seedlings. Polish Journal of Ecology 58, 259–271 (2010).

[b28] Ishii H., Hamada Y. & Utsugi H. Variation in light-intercepting area and photosynthetic rate of sun and shade shoots of two *Picea* species in relation to the angle of incoming light. Tree Physiology 32, 1227–1236 (2012). 23077118 10.1093/treephys/tps090

[b29] Ishii H., Kitaoka S., Fujisaki T., Maruyama Y. & Koike T. Plasticity of shoot and needle morphology and photosynthesis of two *Picea* species with different site preferences in northern Japan. Tree Physiology 27, 1595–1605 (2007). 17669749 10.1093/treephys/27.11.1595

[b30] Hollinger D. Optimality and nitrogen allocation in a tree canopy. Tree Physiology 16, 627–634 (1996). 14871700 10.1093/treephys/16.7.627

[b31] Valio I. Effects of shading and removal of plant parts on growth of *Trema micrantha* seedlings. Tree Physiology 21, 65–70 (2001). 11260826 10.1093/treephys/21.1.65

[b32] Kurepin L. V., Walton L. J., Yeung E. C., Chinnappa C. & Reid D. M. The interaction of light irradiance with ethylene in regulating growth of *Helianthus annuus* shoot tissues. Plant Growth Regulation 62, 43–50 (2010).

[b33] Niinemets Ü. & Kull O. Biomass investment in leaf lamina versus lamina support in relation to growth irradiance and leaf size in temperate deciduous trees. Tree Physiology 19, 349–358 (1999). 12651556 10.1093/treephys/19.6.349

[b34] Stenberg P. Penumbra in within-shoot and between-shoot shading in conifers and its significance for photosynthesis. Ecological Modelling 77, 215–231 (1995).

[b35] Wang G. G., Bauerle W. L. & Mudder B. T. Effects of light acclimation on the photosynthesis, growth, and biomass allocation in American chestnut. Forest Ecology and Management 226, 173–180 (2006).

[b36] Sprugel D. G., Brooks J. R. & Hinckley T. M. Effects of light on shoot geometry and needle morphology in *Abies amabilis*. Tree Physiology 16, 91–98 (1996). 14871751 10.1093/treephys/16.1-2.91

[b37] Beaumont S. & Burns K. C. Vertical gradients in leaf trait diversity in a New Zealand forest. Trees 23, 339–346 (2009).

[b38] Waite M. & Sack L. How does moss photosynthesis relate to leaf and canopy structure? Trait relationships for 10 Hawaiian species of contrasting light habitats. New Phytologist 185, 156–172 (2010). 19863726 10.1111/j.1469-8137.2009.03061.x

[b39] Hallik L., Niinemets Ü. & Wright I. J. Are species shade and drought tolerance reflected in leaf-level structural and functional differentiation in Northern Hemisphere temperate woody flora? New Phytologist 184, 257–274 (2009). 19674334 10.1111/j.1469-8137.2009.02918.x

[b40] Sims D. A., Gebauer R. L. E. & Pearcy R. W. Scaling sun and shade photosynthetic acclimation of *Alocasia macrorrhiza* to whole-plant performance-II. Simulation of carbon balance and growth at different photon flux densities. Plant, Cell & Environment 17, 889–900 (1994).

[b41] Vincent G. Leaf photosynthetic capacity and nitrogen content adjustment to canopy openness in tropical forest tree seedlings. Journal of Tropical Ecology 17, 495–509 (2001).

[b42] Le Roux X., Sinoquet H. & Vandame M. Spatial distribution of leaf dry weight per area and leaf nitrogen concentration in relation to local radiation regime within an isolated tree crown. Tree Physiology 19, 181–188 (1999). 12651581 10.1093/treephys/19.3.181

[b43] Kull O. & Niinemets Ü. Variations in leaf morphometry and nitrogen concentration in *Betula pendula* Roth., *Corylus avellana* L. and *Lonicera xylosteum* L. Tree Physiology 12, 311–318 (1993). 14969921 10.1093/treephys/12.3.311

[b44] Leuning R., Cromer R. & Rance S. Spatial distributions of foliar nitrogen and phosphorus in crowns of *Eucalyptus grandis*. Oecologia 88, 504–510 (1991). 10.1007/BF00317712 28312619

[b45] Niinemets Ü. Distribution of foliar carbon and nitrogen across the canopy of *Fagus sylvatica*: adaptation to a vertical light gradient. Acta Oecologica 16, 525–541 (1995).

[b46] Niinemets Ü. Role of foliar nitrogen in light harvesting and shade tolerance of four temperate deciduous woody species. Functional Ecology 11, 518–531 (1997).

[b47] Niinemets Ü. Acclimation to low irradiance in *Picea abies*: influences of past and present light climate on foliage structure and function. Tree Physiology 17, 723–732 (1997). 14759897 10.1093/treephys/17.11.723

[b48] Niinemets Ü. & Lukjanova A. Total foliar area and average leaf age may be more strongly associated with branching frequency than with leaf longevity in temperate conifers. New Phytologist 158, 75–89 (2003).

[b49] Wright I. J. & Westoby M. Leaves at low versus high rainfall: coordination of structure, lifespan and physiology. New Phytologist 155, 403–416 (2002). 10.1046/j.1469-8137.2002.00479.x 33873314

[b50] Schulze E.-D., Küppers M. & Matyssek R. In On the Economy of Plant Form and Function (ed Givnish) 585–602 (Cambridge University Press, 1986).

[b51] Sprugel D., Hinckley T. & Schaap W. The theory and practice of branch autonomy. Annual Review of Ecology and Systematics 22, 309–334 (1991).

[b52] Suzuki A. Influence of shoot architectural position on shoot growth and branching patterns in *Cleyera japonica*. Tree Physiology 22, 885–889 (2002). 12184978 10.1093/treephys/22.12.885

[b53] Takenaka A. Shoot growth responses to light microenvironment and correlative inhibition in tree seedlings under a forest canopy. Tree Physiology 20, 987–991 (2000). 11303574 10.1093/treephys/20.14.987

[b54] Farque L., Sinoquet H. & Colin F. Canopy structure and light interception in *Quercus petraea* seedlings in relation to light regime and plant density. Tree Physiology 21, 1257–1267 (2001). 11696413 10.1093/treephys/21.17.1257

[b55] Oker-Blom P. Photosynthesis of a Scots pine shoot: simulation of the irradiance distribution and photosynthesis of a shoot in different radiation fields. Agricultural and Forest Meteorology 34, 31–40 (1985).

[b56] Leverenz J. & Hinckley T. Shoot structure, leaf area index and productivity of evergreen conifer stands. Tree Physiology 6, 135–149 (1990). 14972946 10.1093/treephys/6.2.135

[b57] Page V., Blösch R. M. & Feller U. Regulation of shoot growth, root development and manganese allocation in wheat (*Triticum aestivum*) genotypes by light intensity. Plant Growth Regulation 67, 209–215 (2012).

[b58] Cheng X., Han H., Kang F., Song Y. & Liu K. Variation in biomass and carbon storage by stand age in pine (*Pinus tabulaeformis*) planted ecosystem in Mt. Taiyue, Shanxi, China. Journal of Plant Interactions 9, 521–528 (2014).

[b59] Wright I. J. . The worldwide leaf economics spectrum. Nature 428, 821–827 (2004). 15103368 10.1038/nature02403

